# Supplementary material for: T cell receptor clonotypes predict human leukocyte antigen allele carriage and antigen exposure history
Source: Commun Biol. 2026 Jan 13;9:50. doi: 10.1038/s42003-025-09140-2 (PMC12800166; doi:10.1038/s42003-025-09140-2)
Supplement: Supplementary file 2 — Supplementary Information [file 42003_2025_9140_MOESM2_ESM.pdf]

Supplementary figures

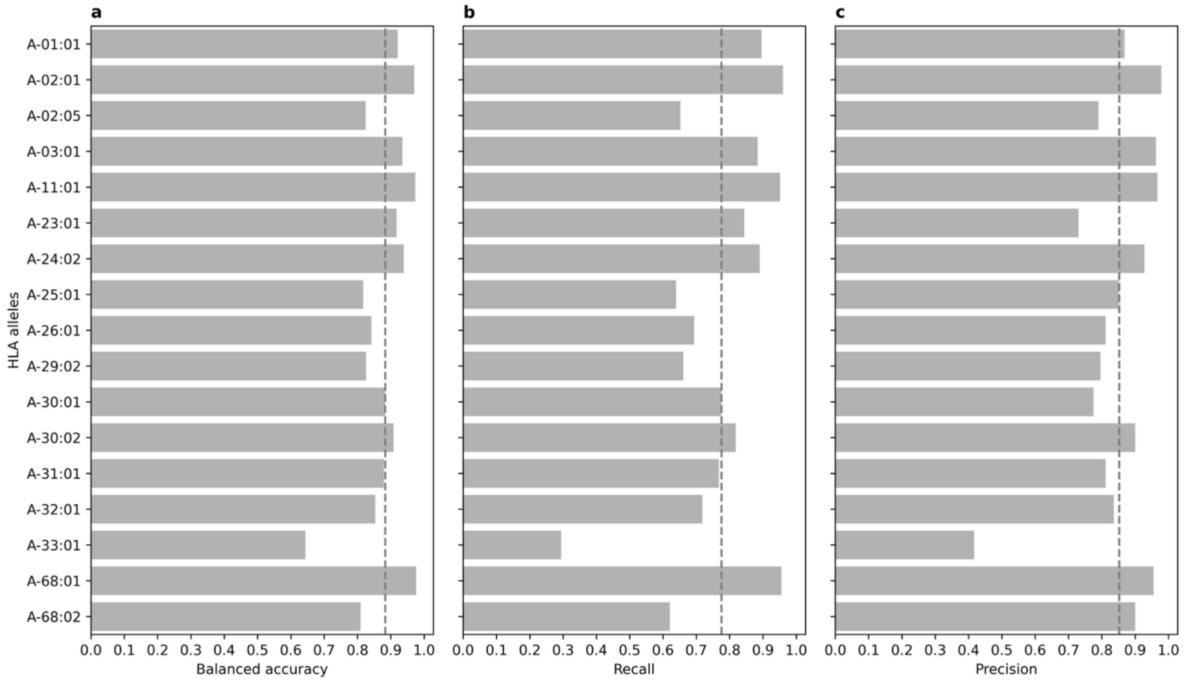

Supplementary Figure 1: *The performance of models to predict the HLA-A allele-carriership status from the TRB repertoire evaluated on a test dataset made of 1,111 repertoires.* On the y-axis the models of each *HLA-A* allele are shown, for example, A-01:01 represents a model that predicts whether an individual is a carrier for the HLA-A*01:01 allele or not. (**a**), (**b**) and (**c**) depict the balanced accuracy, recall and precision, respectively. Across all panels, grey dashed lines represent the median.

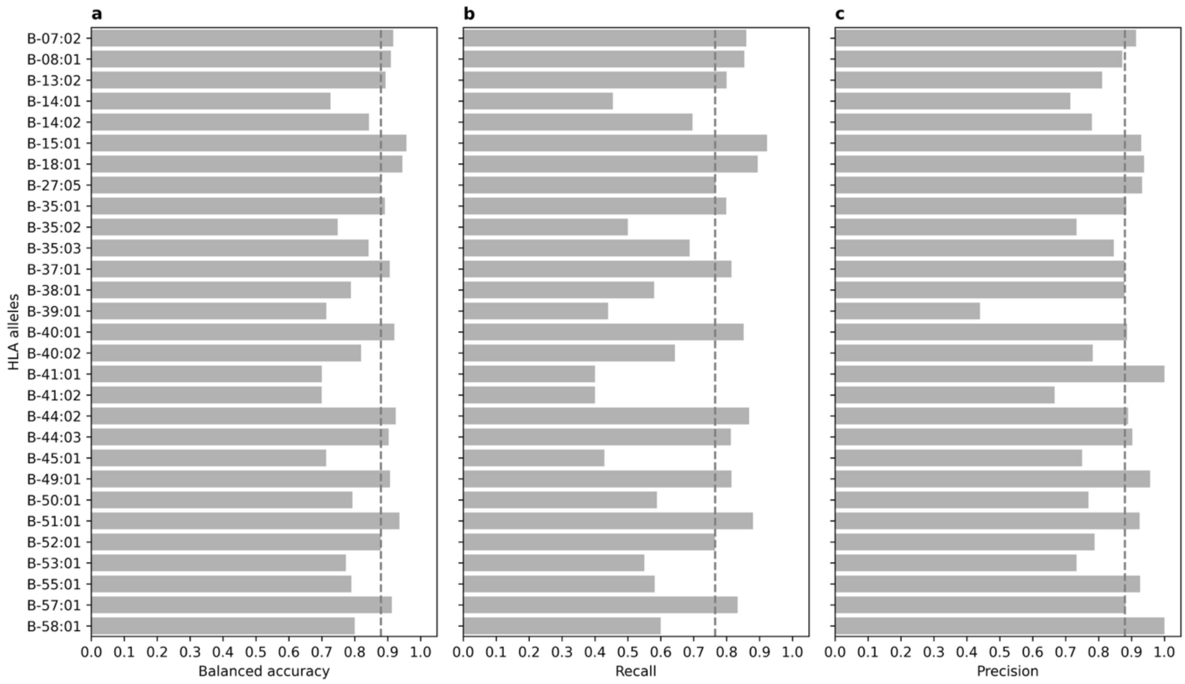

Supplementary Figure 2: *The performance of models to predict HLA-B allele-carriership status from the TRB repertoire evaluated on a test dataset made of 1,111 repertoires*. On the y-axis the models of each *HLA-B* allele are shown, for example, B-07:02 represents a model that predicts whether an individual is a carrier for the HLA-B*07:02 allele or not. (**a**), (**b**) and (**c**), depict the balanced accuracy, recall and precision, respectively. Across all panels, grey dashed lines represent the median.

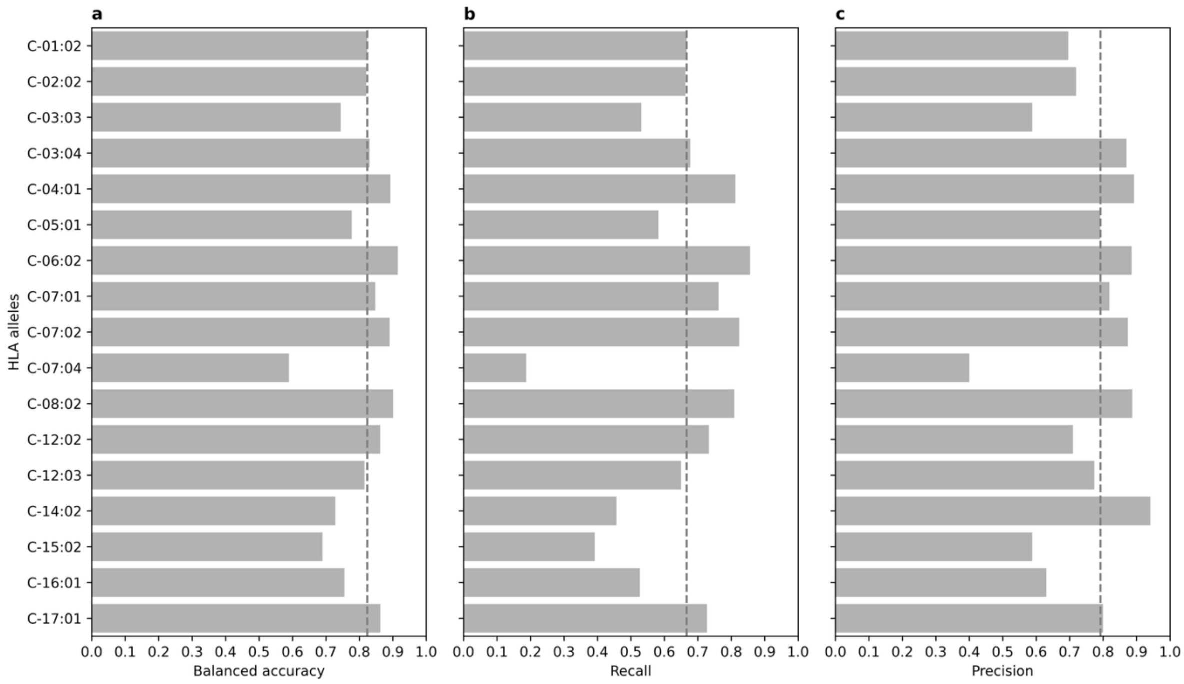

Supplementary Figure 3: *The performance of models to predict HLA-C allele-carriership status from the TRB repertoire evaluated on a test dataset made of 1,111 repertoires*. On the y-axis the models of each *HLA-C* allele are shown, for example, C-01:02 represents a model that predicts whether an individual is a carrier for the HLA-C*01:02 allele or not. (**a**), (**b**) and (**c**), depict the balanced accuracy, recall and precision, respectively. Across all panels, grey dashed lines represent the median.

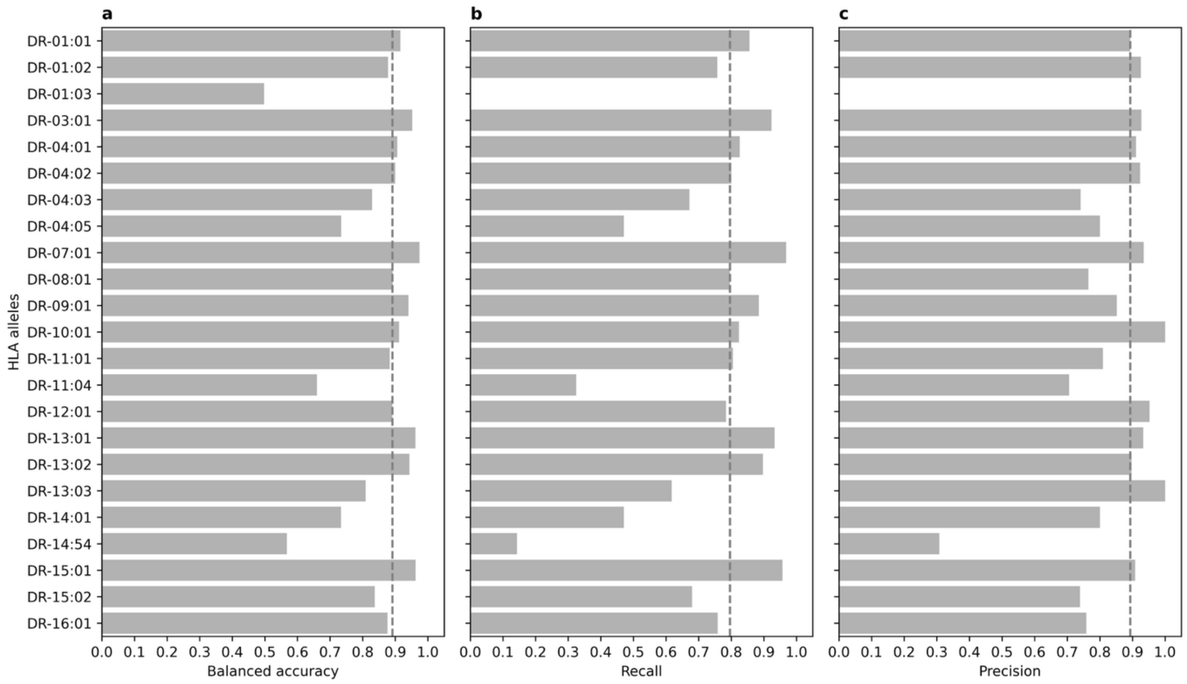

Supplementary Figure 4: *The performance of models to predict HLA-DR allele-carriership status from the TRB repertoire evaluated on a test dataset made of 1,111 repertoires*. On the y-axis the models of each *HLA-D*R allele are shown, for example, DR-01:01 represents a model that predicts whether an individual is a carrier for the HLA-DRB1*01:01 allele or not. (**a**), (**b**) and (**c**), depict the balanced accuracy, recall and precision, respectively. Across all panels, grey dashed lines represent the median.

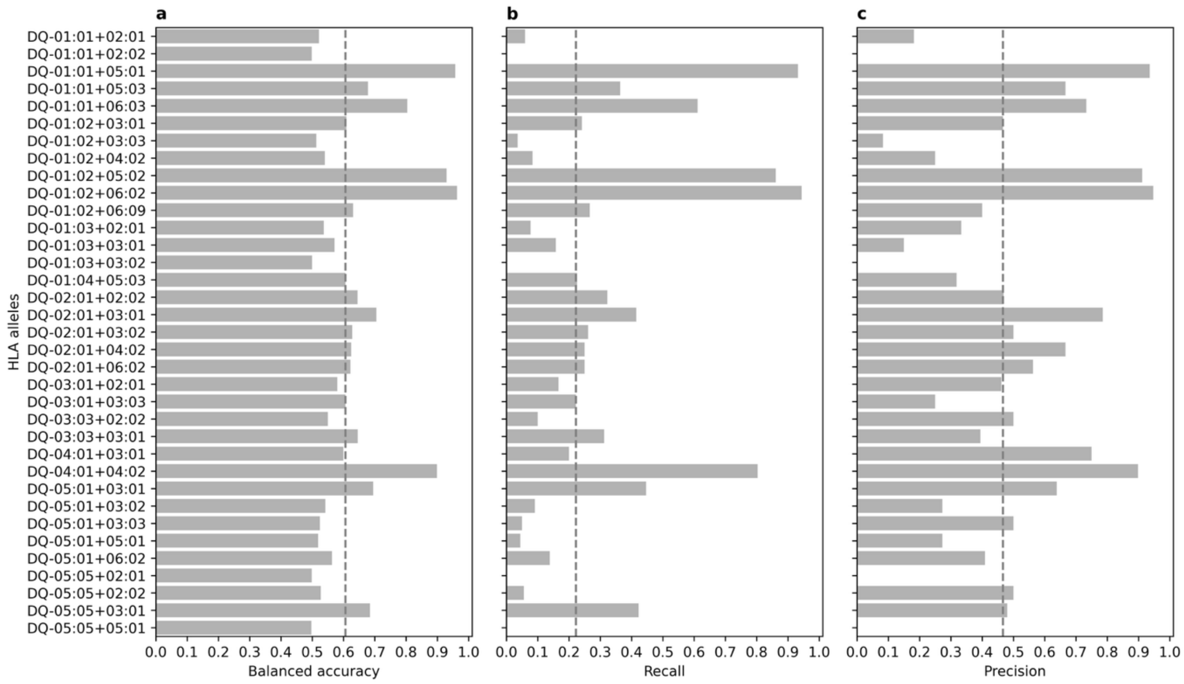

Supplementary Figure 5: *The performance of models to predict HLA-DQ allele-carriership status from the TRB repertoire evaluated on a test dataset made of 1,111 repertoires*. On the y-axis the models of each *HLA-DQ* allele are shown, for example, DR-01:01+02:01 represents a model that predicts whether an individual is a carrier for the HLA-DQA*01:01-DQB1*02:01 allele or not. (**a**), (**b**) and (**c**), depict the balanced accuracy, recall and precision, respectively. Across all panels, grey dashed lines represent the median.

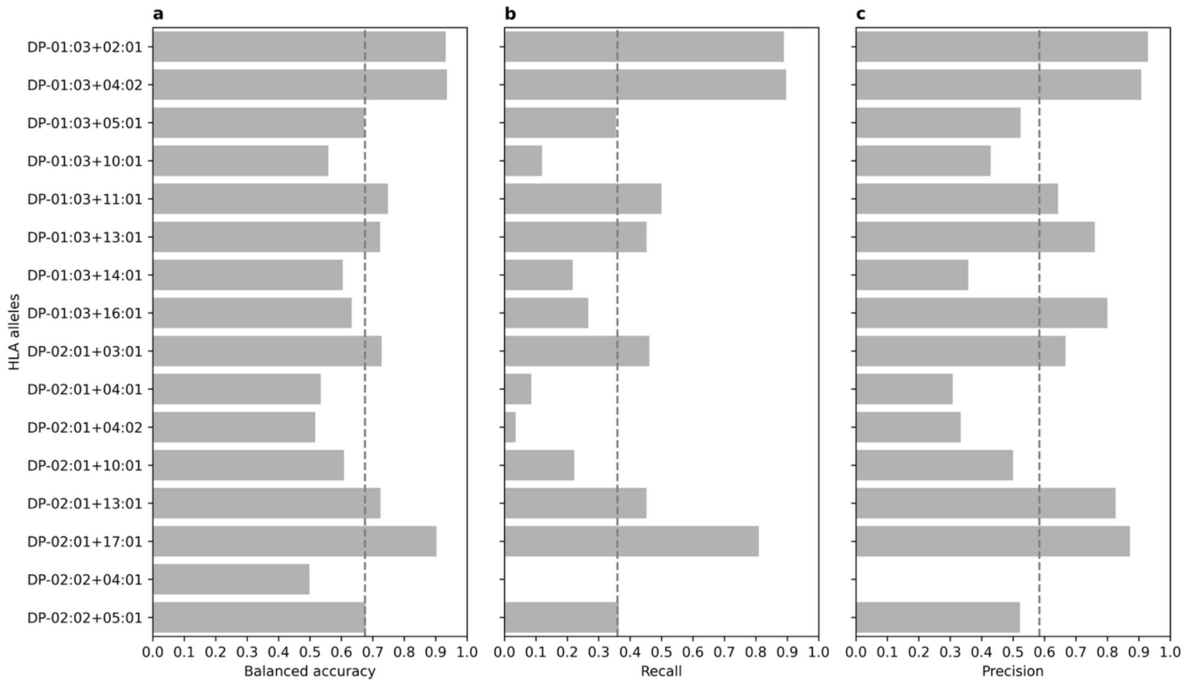

Supplementary Figure 6: *The performance of models to predict HLA-DP allele-carriership status from the TRB repertoire evaluated on a test dataset made of 1,111 repertoires*. On the y-axis the models of each *HLA-DP* allele are shown, for example, DP-01:03+02:01 represents a model that predicts whether an individual is a carrier for the HLA-DPA*01:03-DPB1*02:01 allele or not. (**a**), (**b**) and (**c**), depict the balanced accuracy, recall and precision, respectively. Across all panels, grey dashed lines represent the median.

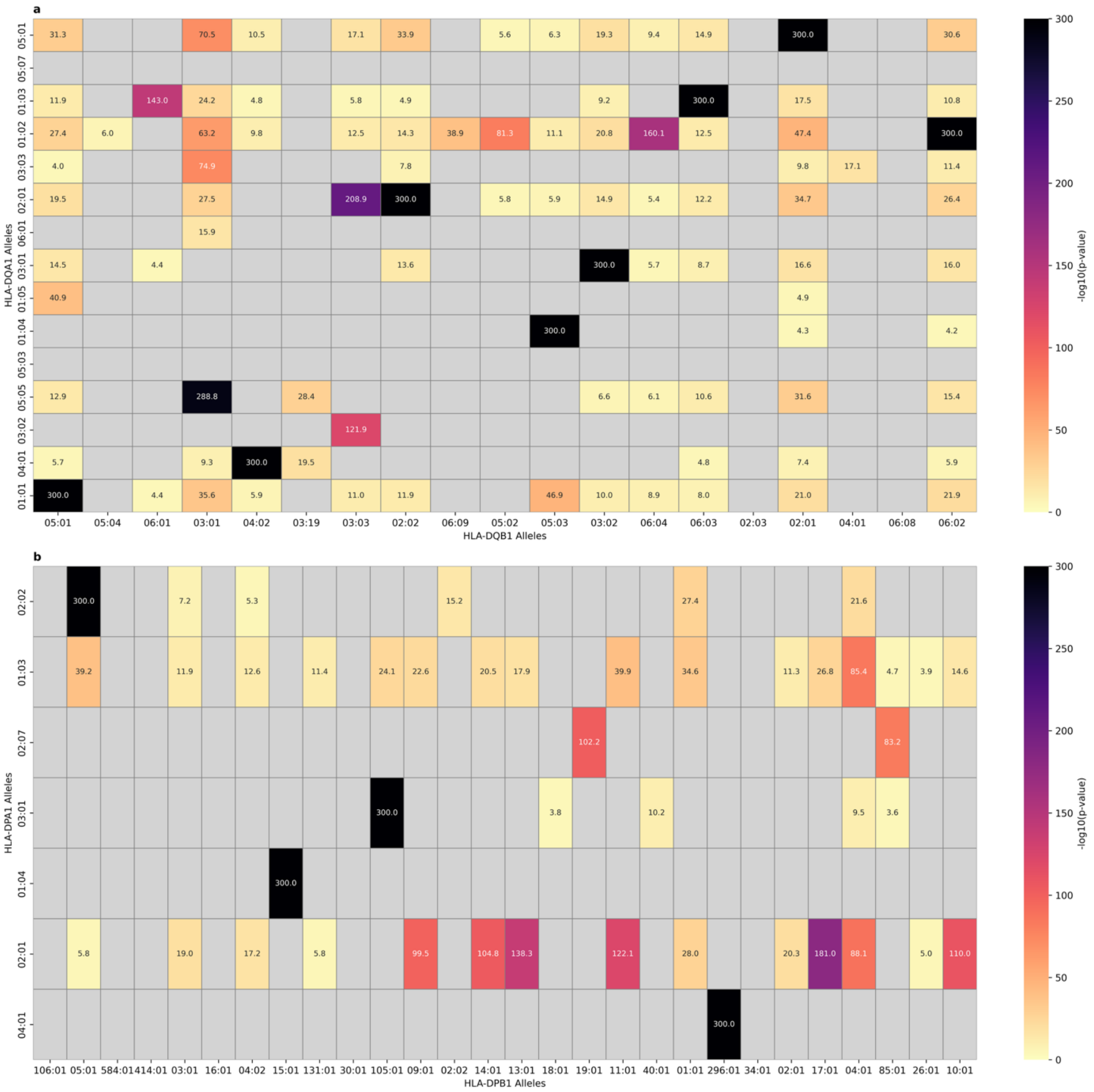

Supplementary Figure 7: *The identification of cis and trans HLA-DQ (**a**) and HLA-DP (**b**) complexes by comparing their observed frequencies to their expected carriership frequencies using a chi-square test*. We used Bonferroni correction to correct for multiple testing, where Trans complexes are depicted in grey and potential cis complexes, (chi-square P-value > Bonferroni-corrected threshold) are shown in a color-gradient representing their association P-value in both panels.

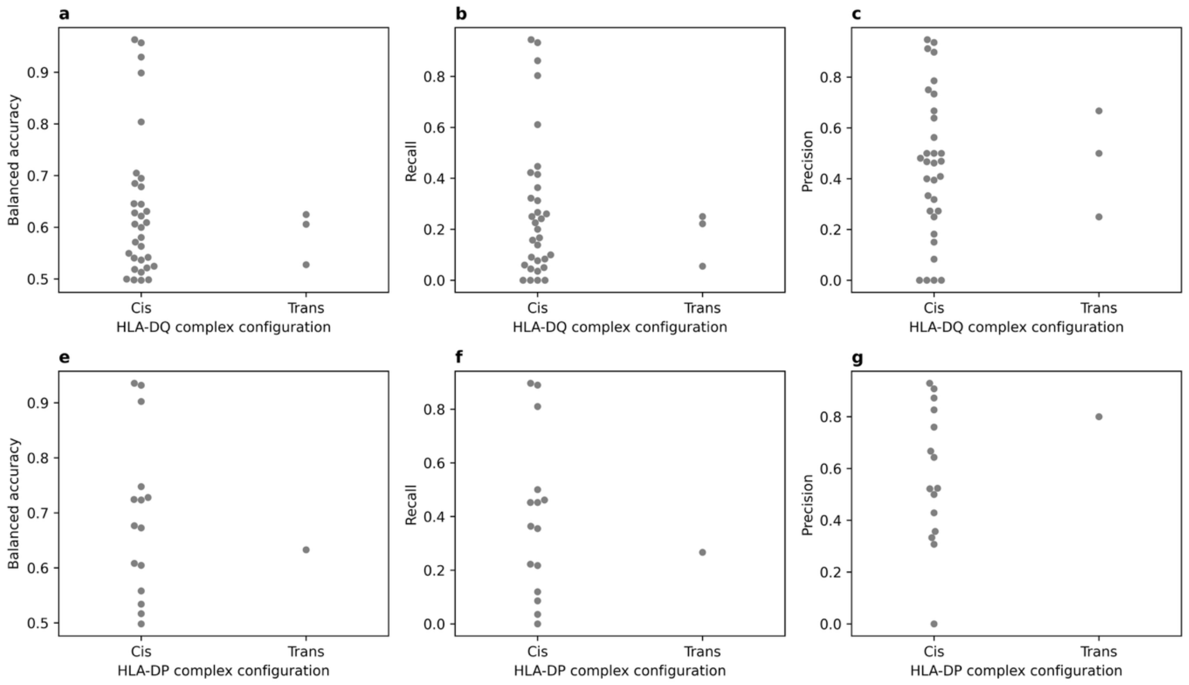

Supplementary Figure 8: *The performance of cis and trans HLA-DQ and HLA-DP complexes models on the validation dataset.* (**a**), (**b**) and (**c**) depict the balanced accuracy, recall and precision of cis and trans HLA-DQ complexes while (**e**), (**f**) and (**g**), shows the same performance metrics across cis and trans HLA-DP complexes.

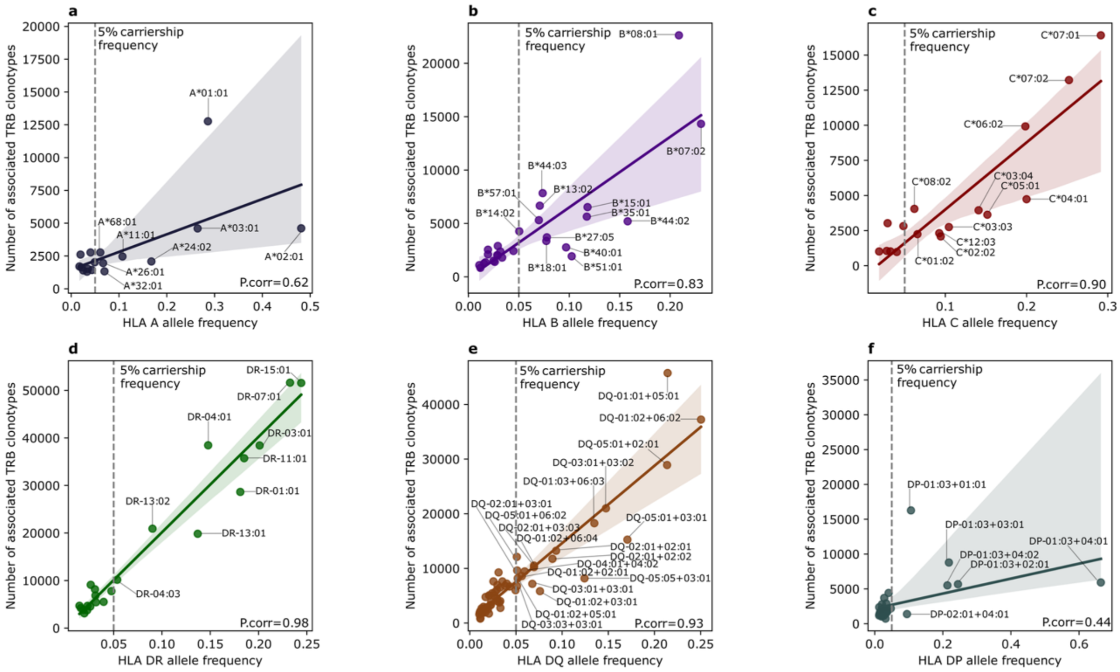

Supplementary Figure 9: *The relationship between HLA allele carriership frequency (shown on the x-axis) and the number of associated TRB clonotypes (shown on the y-axis) for the six classical HLA complexes. HLA-A* (**a**), *HLA-B* (**b**), *HLA-C* (**c**), *HLA-DR* (**d**), *HLA-DQ* (**e**), *HLA-DP* (**f**). Lastly, P.corr donates the Pearson correlation co-efficiency.

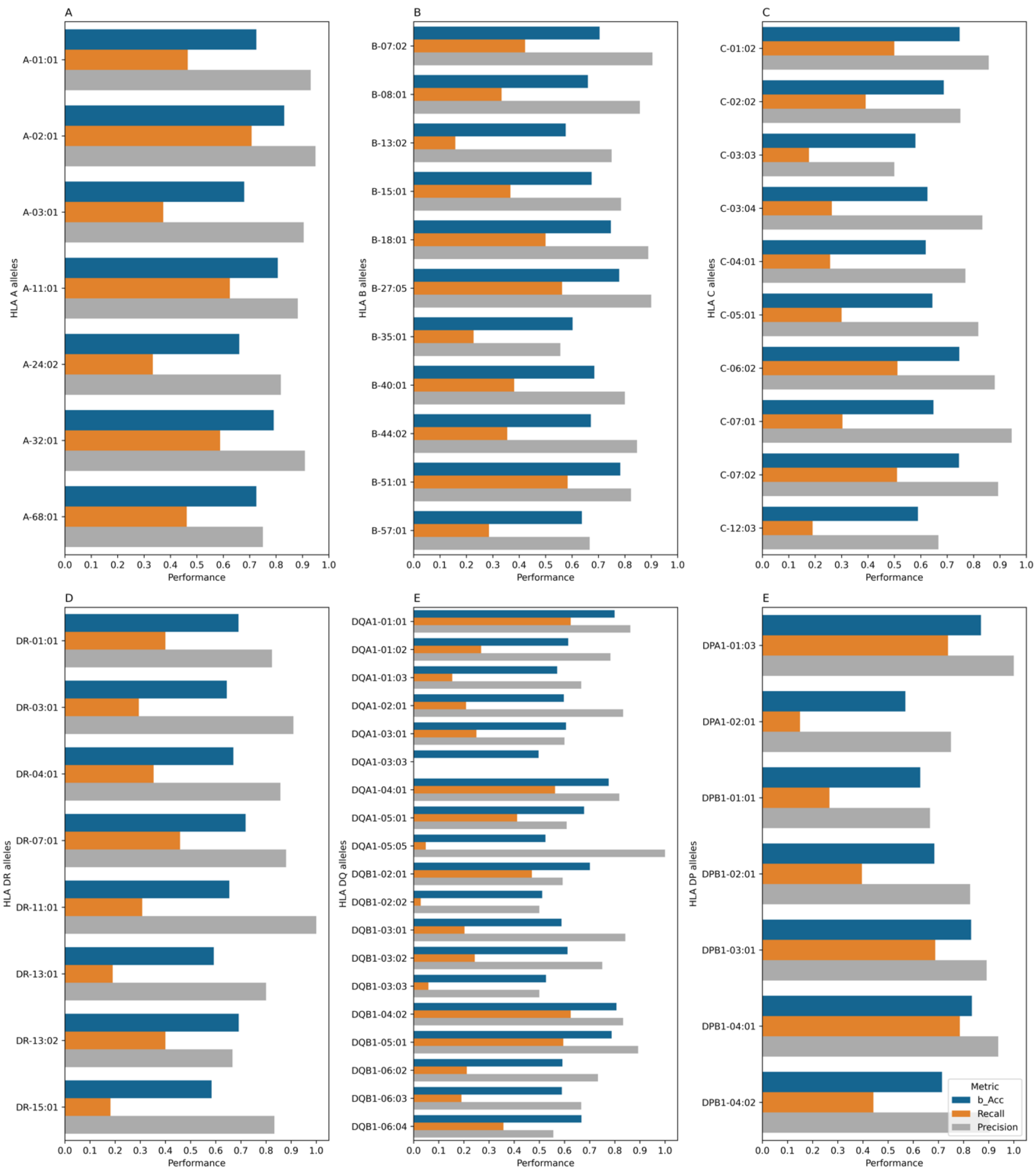

Supplementary Figure 10: *The performance of the TRB-based HLA imputation models on an independent test dataset obtained from Rosati et al.*[1]. (**A**) shows the performance of *HLA-A* allele models, while (**B**), (**C**), and (**D**) show the performance of the *HLA-B*, *HLA-C* and *HLA-DR* alleles, respectively. (**E**) and (**F**) show the performance of *HLA-DQA*/*DQB* and *HLA-DPA*/*DPB* proteins, respectively. We have not measured the imputation performance at the functional αβ HLA complex level for *HLA-DQ* and *HLA-DP* alleles, given that some of the *HLA-DQ* and *HLA-DP* alleles were not typed at the four-digit resolution in the *Rosati et al.*[1] dataset. Across all panels, alleles with carriership frequency <5% (n<12 samples) were excluded from the analysis.

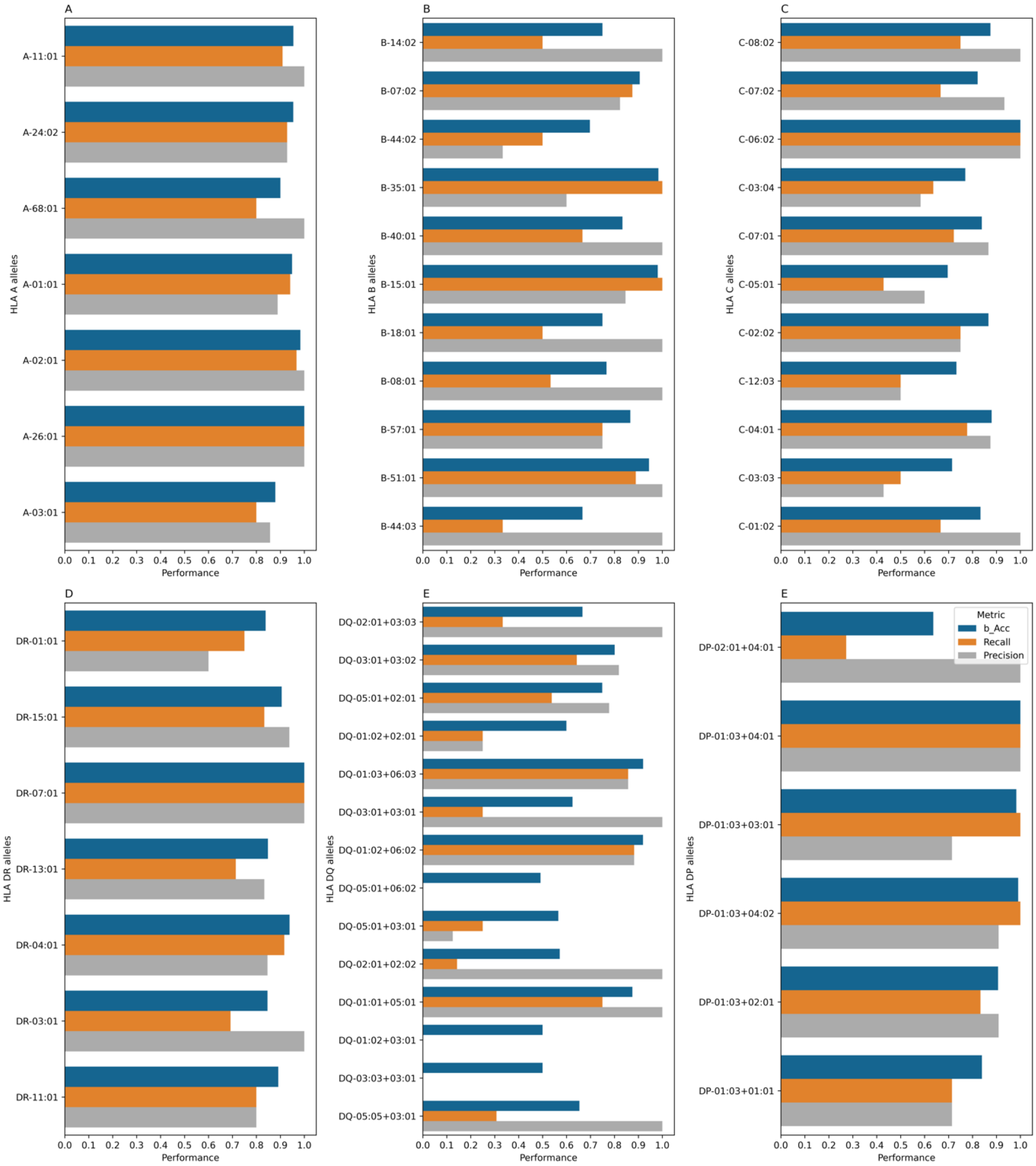

Supplementary Figure 11: *The performance of the TRB-based HLA imputation models on an independent test dataset obtained from the immuneCODE dataset*[2]*.* (**A**-**E**) show the performance of *HLA-A*, *HLA-B*, *HLA-C*, *HLA-DR*, *HLA-DQ*, and *HLA-DP* alleles, respectively. Across all panels, alleles with carriership frequency <5% (n<3 samples) were excluded from the analysis.

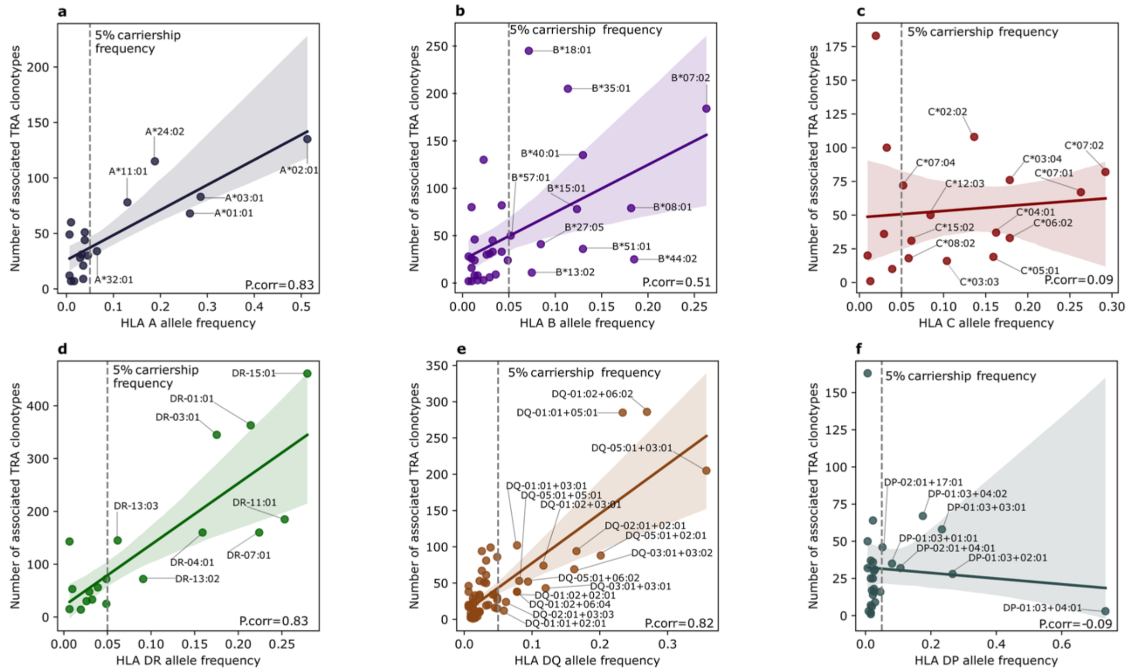

Supplementary Figure 12: *The relationship between HLA allele carriership frequency (shown on the x-axis) and the number of associated TRA clonotypes (shown on the y-axis) for the six classical HLA loci*. *HLA-A* (**a**), *HLA-B* (**b**), *HLA-C* (**c**), *HLA-DR* (**d**), *HLA-DQ* (**e**), *HLA-DP* (**f**). Lastly, P.corr donates the Pearson correlation co-efficiency.

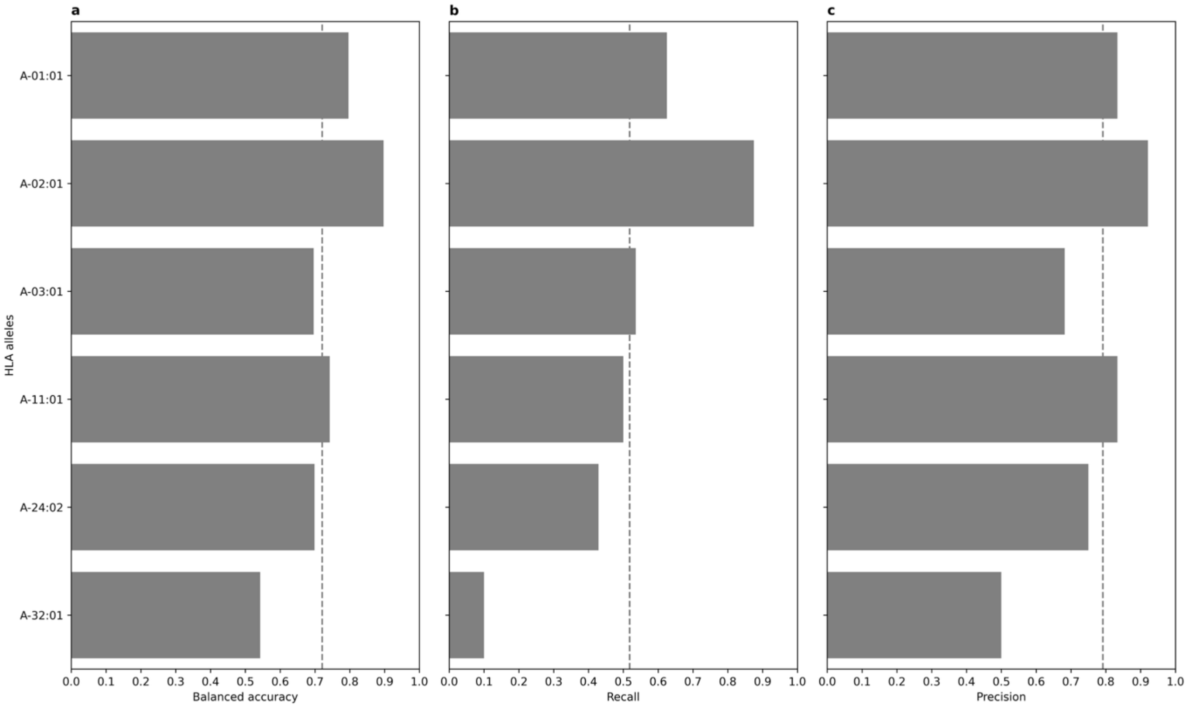

Supplementary Figure 13: *The performance of models to predict the HLA-A allele-carriership status from the TRA repertoire evaluated on a test dataset made of 77 repertoires*. These models were trained on a discovery dataset composite of 308 TRA repertoires with matching HLA calls. On the y-axis the models of each *HLA-A* allele are shown, for example, A-01:01 represents a model that predicts whether an individual is a carrier for the HLA-A*01:01 allele or not. (**a**), (**b**) and (**c**), depict the balanced accuracy, recall and precision, respectively. Across all panels, grey dashed lines represent the median.

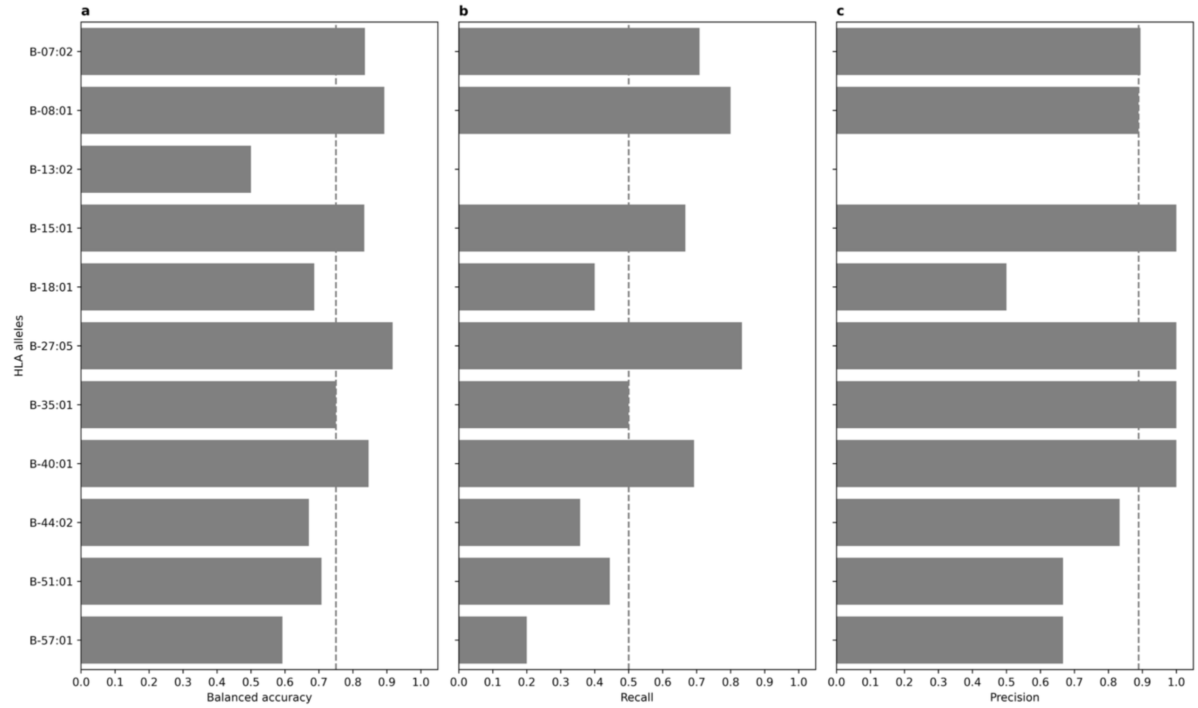

Supplementary Figure 14: *The performance of models to predict the HLA-B allele-carriership status from the TRA repertoire evaluated on a test dataset made of 77 repertoires*. These models were trained on a discovery dataset composite of 308 TRA repertoires with matching HLA calls. On the y-axis the models of each *HLA-B* allele are shown, for example, B*07:02 represents a model that predicts whether an individual is a carrier for the HLA-B*07:02 allele or not. (**a**), (**b**) and (**c**), depict the balanced accuracy, recall and precision, respectively. Across all panels, grey dashed lines represent the median.

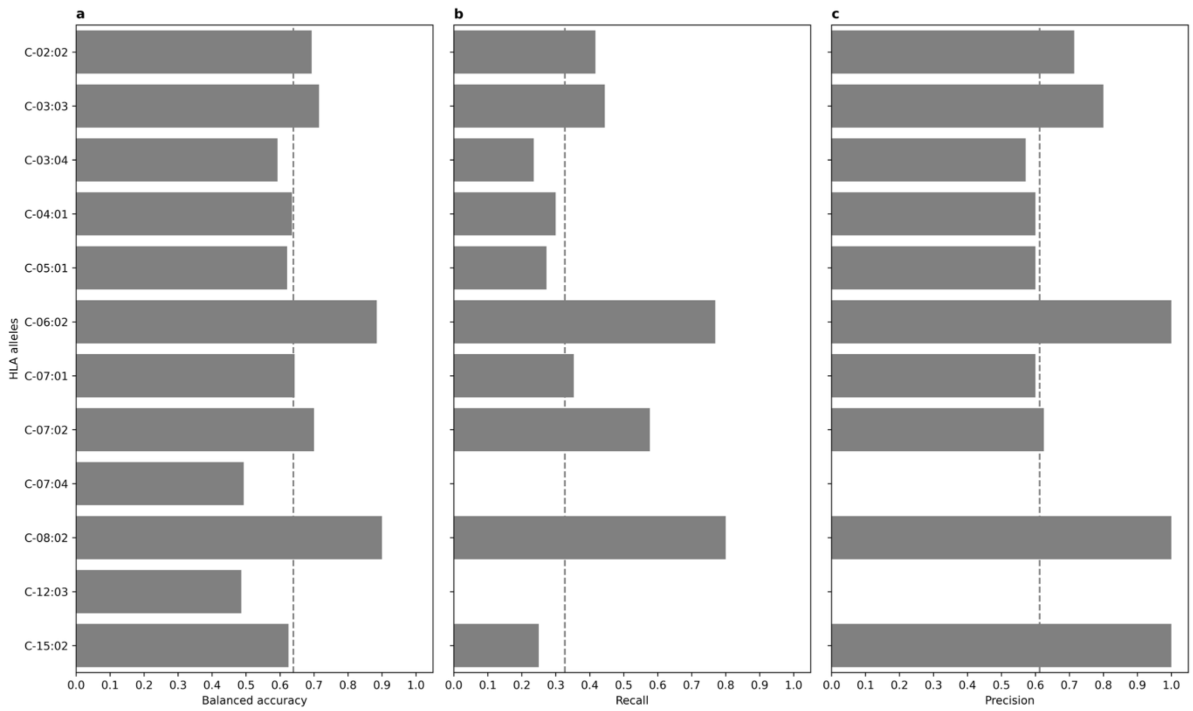

Supplementary Figure 15: *The performance of models to predict the HLA-C allele-carriership status from the TRA repertoire evaluated on a test dataset made of 77 repertoires.* These models were trained on a discovery dataset composite of 308 TRA repertoires with matching HLA calls. On the y-axis the models of each *HLA-C* allele are shown, for example, C*02:02 represents a model that predicts whether an individual is a carrier for the HLA-C*02:02 allele or not. (**a**), (**b**) and (**c**), depict the balanced accuracy, recall and precision, respectively. Across all panels, grey dashed lines represent the median.

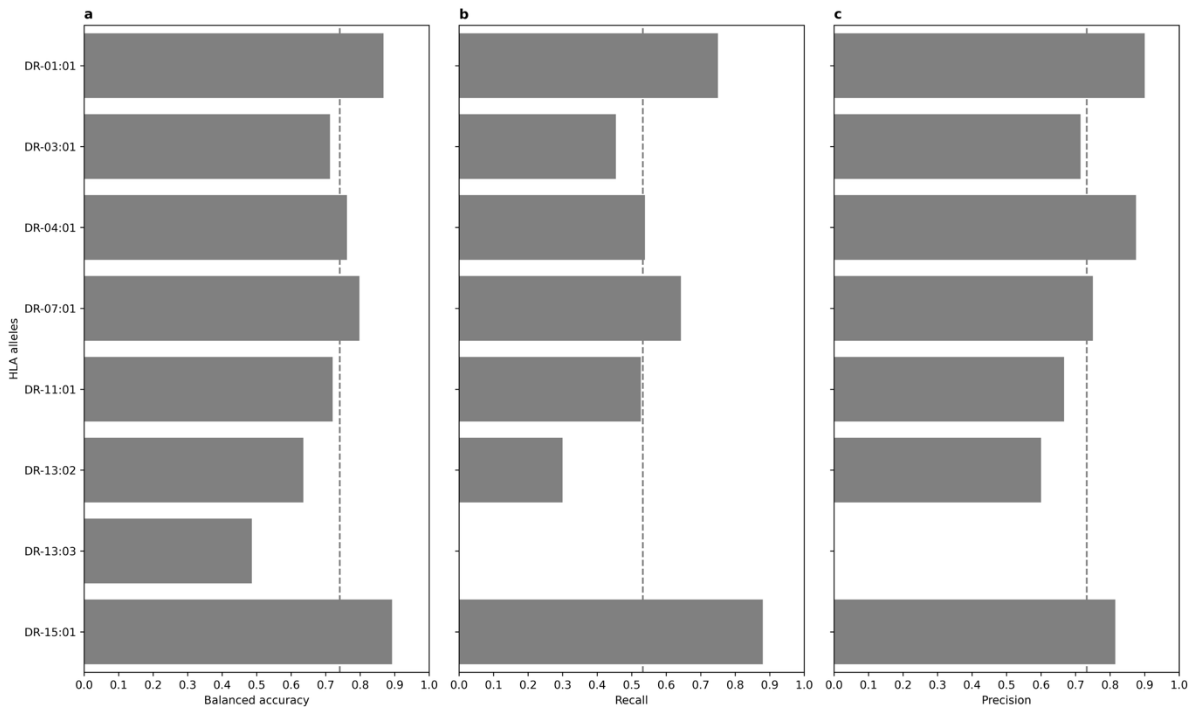

Supplementary Figure 16: *The performance of models to predict the HLA-DR allele-carriership status from the TRA repertoire evaluated on a test dataset made of 77 repertoires.* These models were trained on a discovery dataset composite of 308 TRA repertoires with matching HLA calls. On the y-axis the models of each *HLA-DR* allele are shown, for example, DR*01:01 represents a model that predicts whether an individual is a carrier for the HLA-DRB1*01:01 allele or not. (**a**), (**b**) and (**c**), depict the balanced accuracy, recall and precision, respectively. Across all panels, grey dashed lines represent the median.

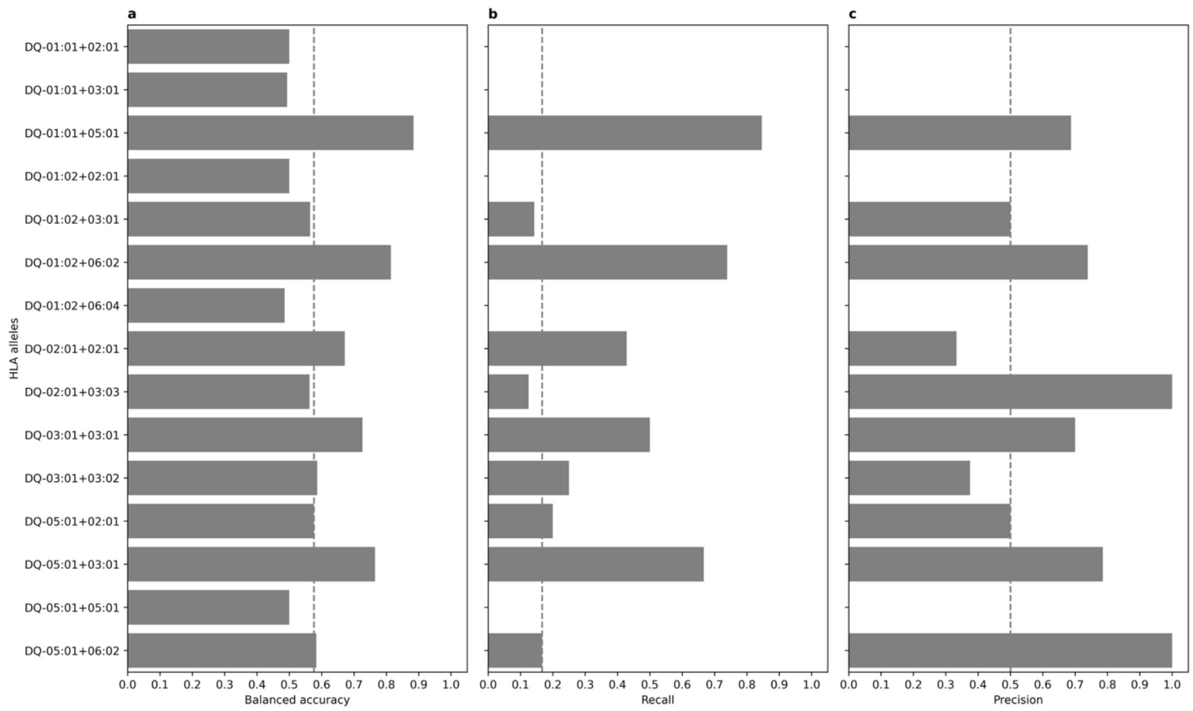

Supplementary Figure 17: *The performance of models to predict the HLA-DQ allele-carriership status from the TRA repertoire evaluated on a test dataset made of 77 repertoires*. These models were trained on a discovery dataset composite of 308 TRA repertoires with matching HLA calls. On the y-axis the models of each *HLA-DQ* allele are shown, for example, DQ*01:01+02:01 represents a model that predicts whether an individual is a carrier for the HLA-DQA*01:01-DQB1*02:01 allele or not. (**a**), (**b**) and (**c**), depict the balanced accuracy, recall and precision, respectively. Across all panels, grey dashed lines represent the median.

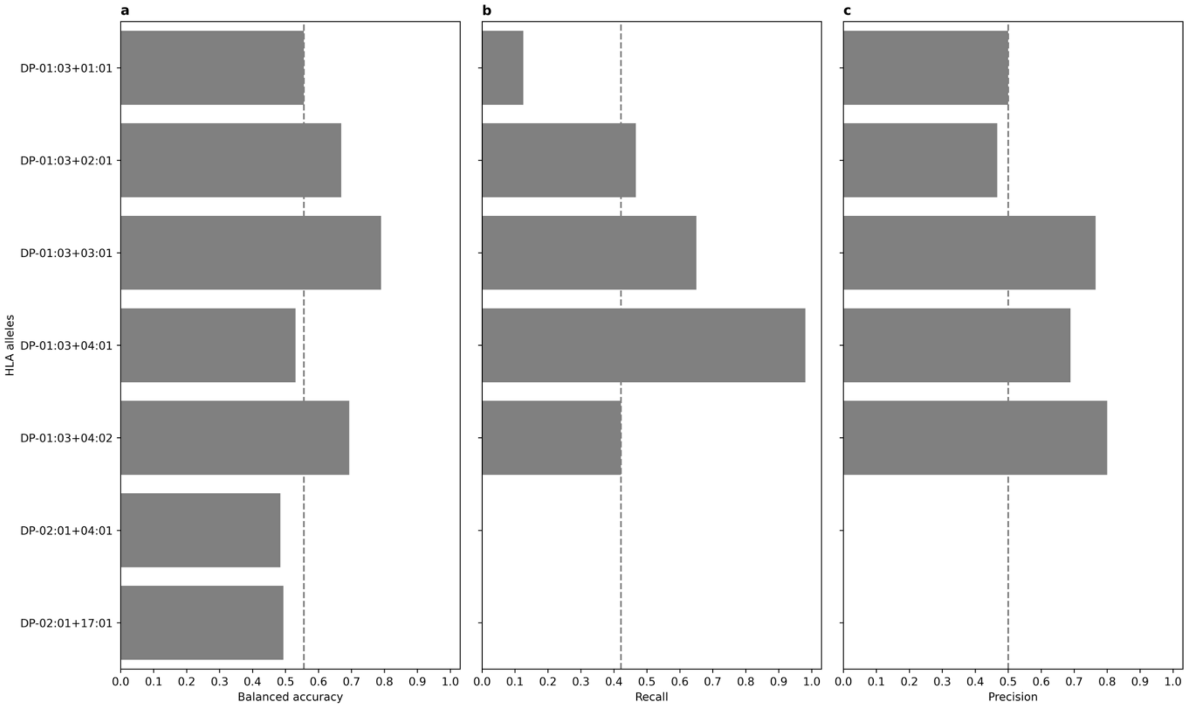

Supplementary Figure 18: *The performance of models to predict the HLA-DP allele-carriership status from the TRA repertoire evaluated on a test dataset made of 77 repertoires*. These models were trained on a discovery dataset composite of 308 TRA repertoires with matching HLA calls. On the y-axis the models of each *HLA-DP* allele are shown, for example, DPA1*01:03+01:01 represents a model that predicts whether an individual is a carrier for the HLA-DPA1*03:01-DPB1*01:01 allele or not. (**a**), (**b**) and (**c**), depict the balanced accuracy, recall and precision, respectively. Across all panels, grey dashed lines represent the median.

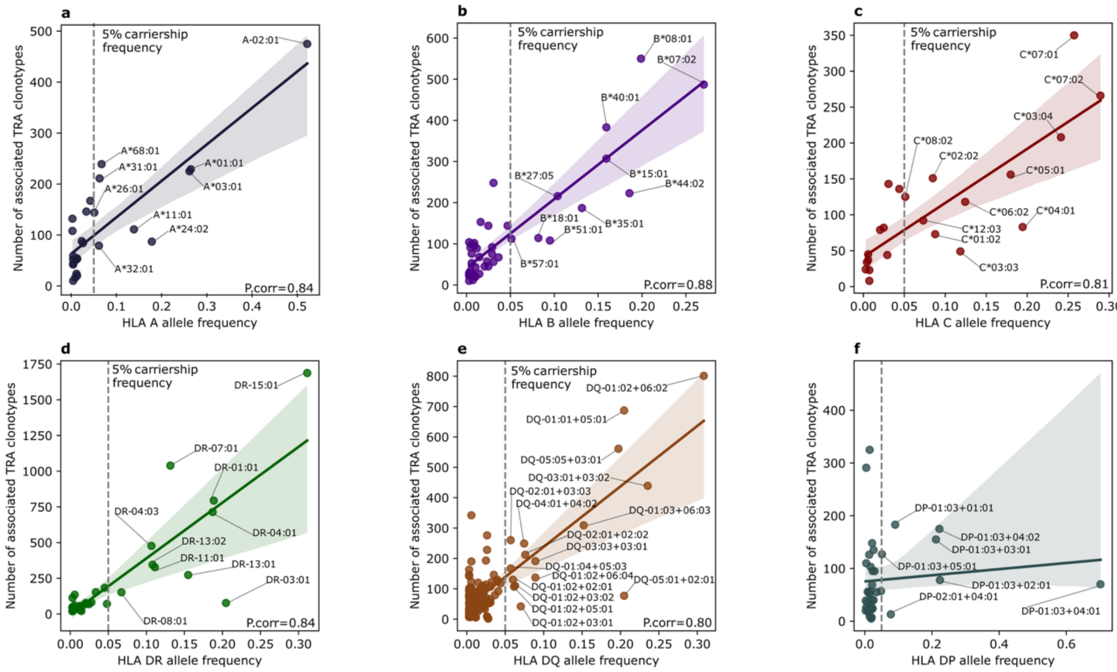

Supplementary Figure 19: *The relationship between HLA allele carriership frequency (shown on the x-axis) and the number of associated TRA clonotypes (shown on the y-axis) for the six classical HLA proteins. HLA-A* (**a**), *HLA-B* (**b**), *HLA-C* (**c**), *HLA-DR* (**d**), *HLA-DQ* (**e**), *HLA-DP* (**f**). Lastly, P.corr donates the Pearson correlation co-efficiency.

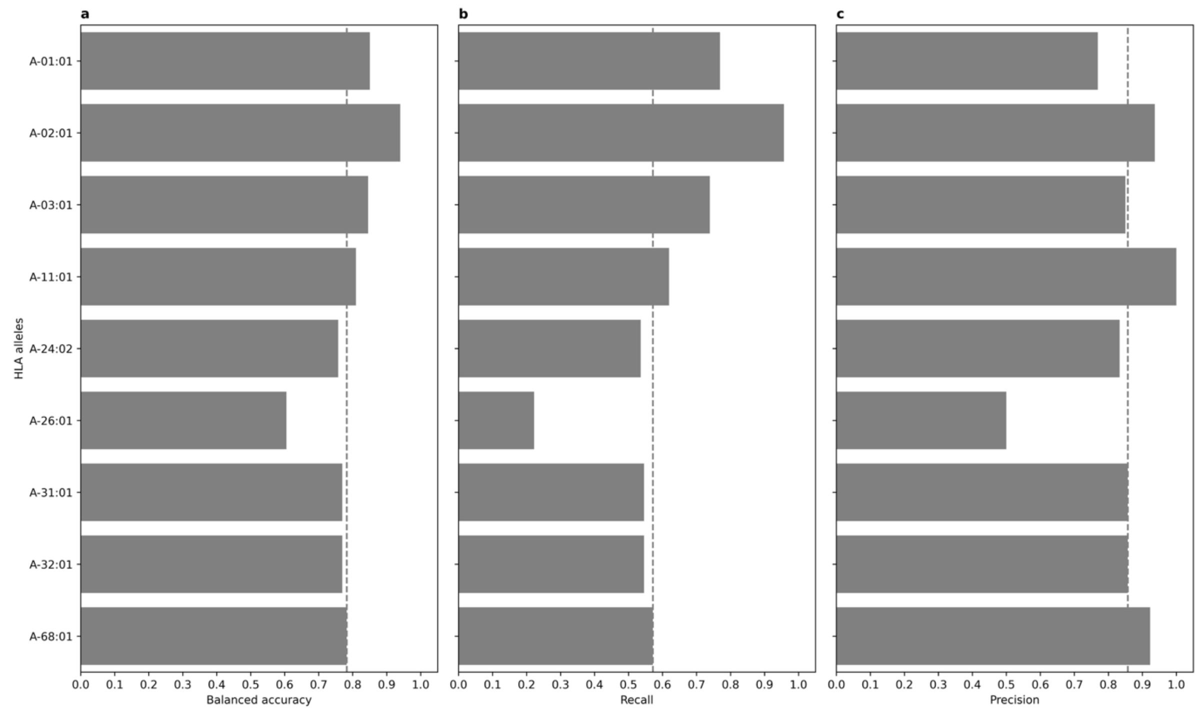

Supplementary Figure 20: *The performance of models to predict the HLA-A allele-carriership status from the TRA repertoire evaluated on a test dataset made of 171repertoires*. These models were trained on a discovery dataset composite of 684 TRA repertoires with matching HLA calls. On the y-axis the models of each *HLA-A* allele are shown, for example, A-01:01 represents a model that predicts whether an individual is a carrier for the HLA-A*01:01 allele or not. (**a**), (**b**) and (**c**), depict the balanced accuracy, recall and precision, respectively. Across all panels, grey dashed lines represent the median.

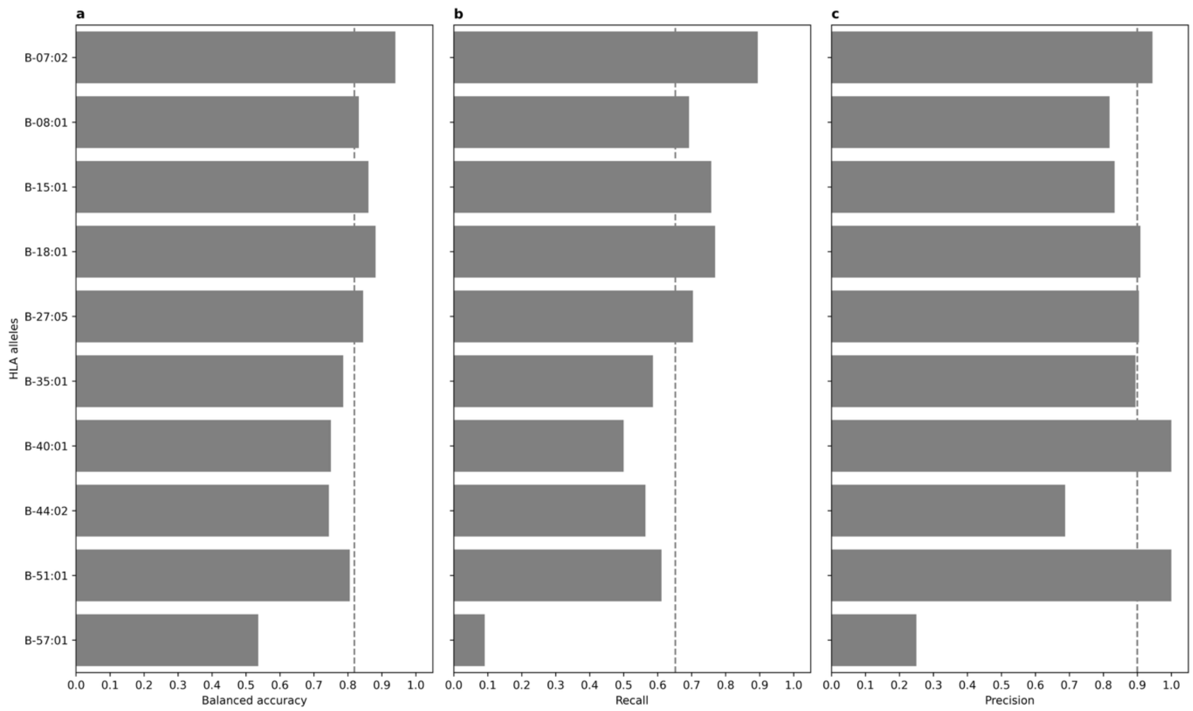

Supplementary Figure 21: *The performance of models to predict the HLA-B allele-carriership status from the TRA repertoire evaluated on a test dataset made of 171 repertoires.* These models were trained on a discovery dataset composite of 684 TRA repertoires with matching HLA calls. On the y-axis the models of each *HLA-B* allele are shown, for example, B-07:02 represents a model that predicts whether an individual is a carrier for the HLA-B*07:02 allele or not. (**a**), (**b**) and (**c**), depict the balanced accuracy, recall and precision, respectively. Across all panels, grey dashed lines represent the median.

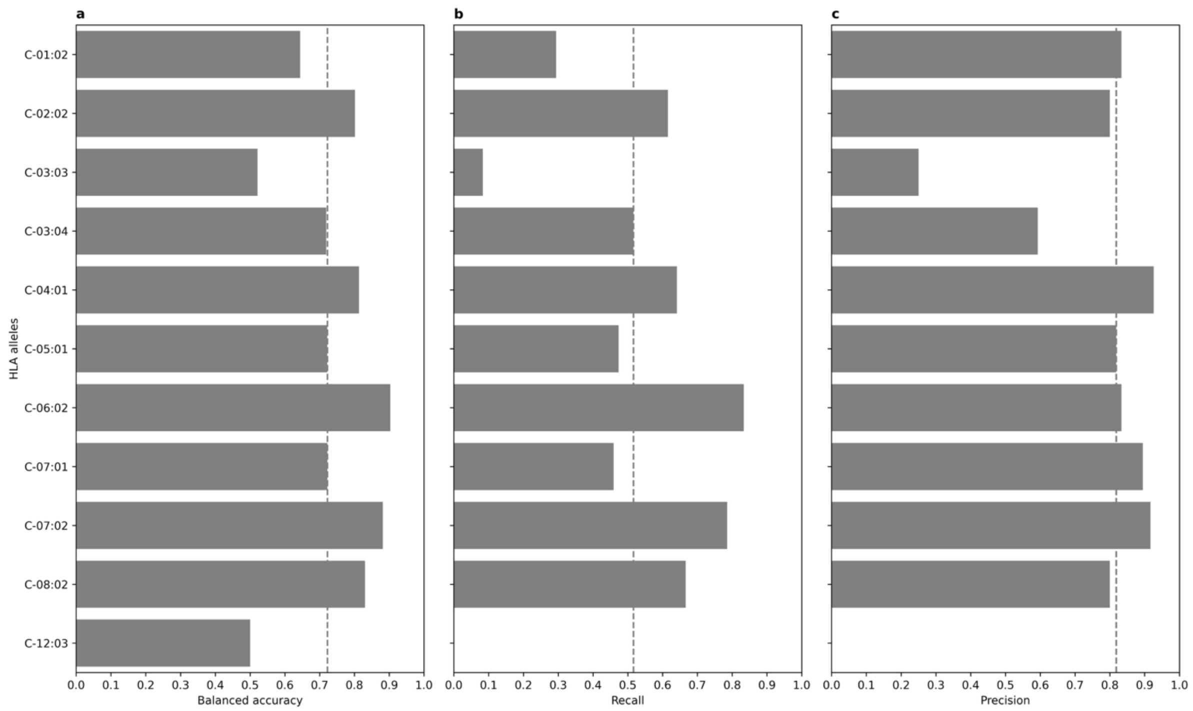

Supplementary Figure 22: *The performance of models to predict the HLA-C allele-carriership status from the TRA repertoire evaluated on a test dataset made of 171 repertoires*. These models were trained on a discovery dataset composite of 684 TRA repertoires with matching HLA calls. On the y-axis the models of each HLA-C allele are shown, for example, C-02:02 represents a model that predicts whether an individual is a carrier for the HLA-C*02:02 allele or not. (**a**), (**b**) and (**c**), depict the balanced accuracy, recall and precision, respectively. Across all panels, grey dashed lines represent the median.

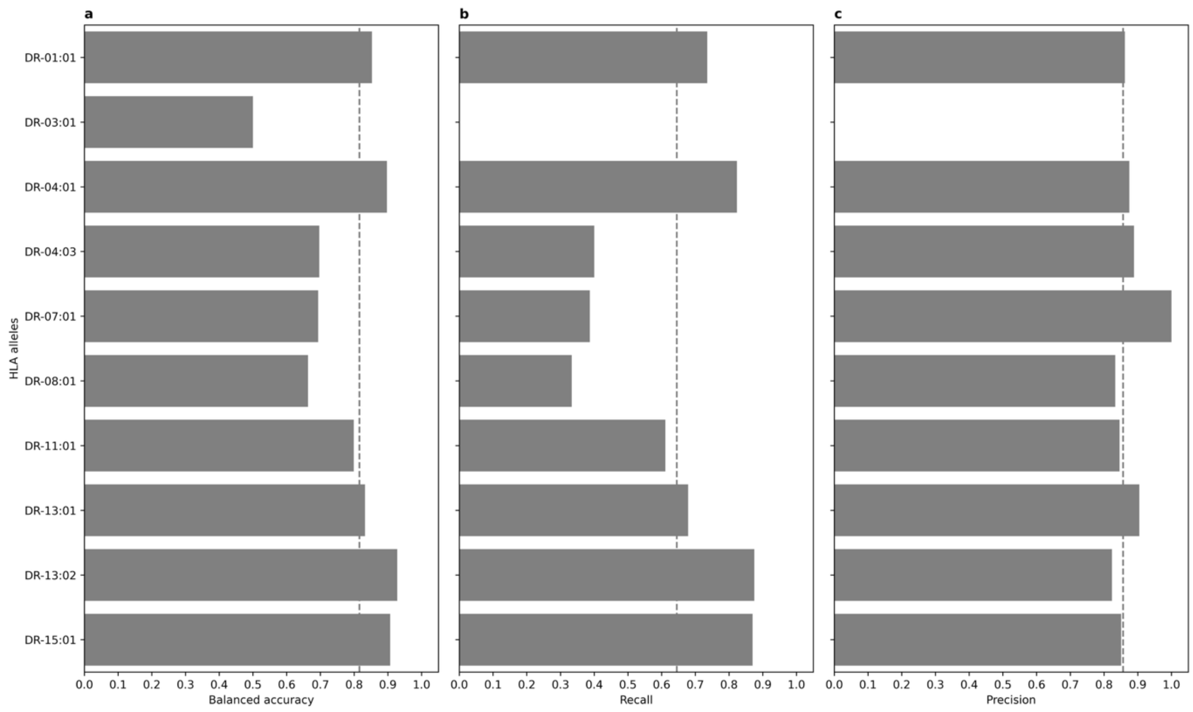

Supplementary Figure 23: *The performance of models to predict the HLA-DR allele-carriership status from the TRA repertoire evaluated on a test dataset made of 171 repertoires*. These models were trained on a discovery dataset composite of 684 TRA repertoires with matching HLA calls. On the y-axis the models of each *HLA-DR* allele are shown, for example, DR-01:01 represents a model that predicts whether an individual is a carrier for the HLA-DRB1*01:01 allele or not. (**a**), (**b**) and (**c**), depict the balanced accuracy, recall and precision, respectively. Across all panels, grey dashed lines represent the median.

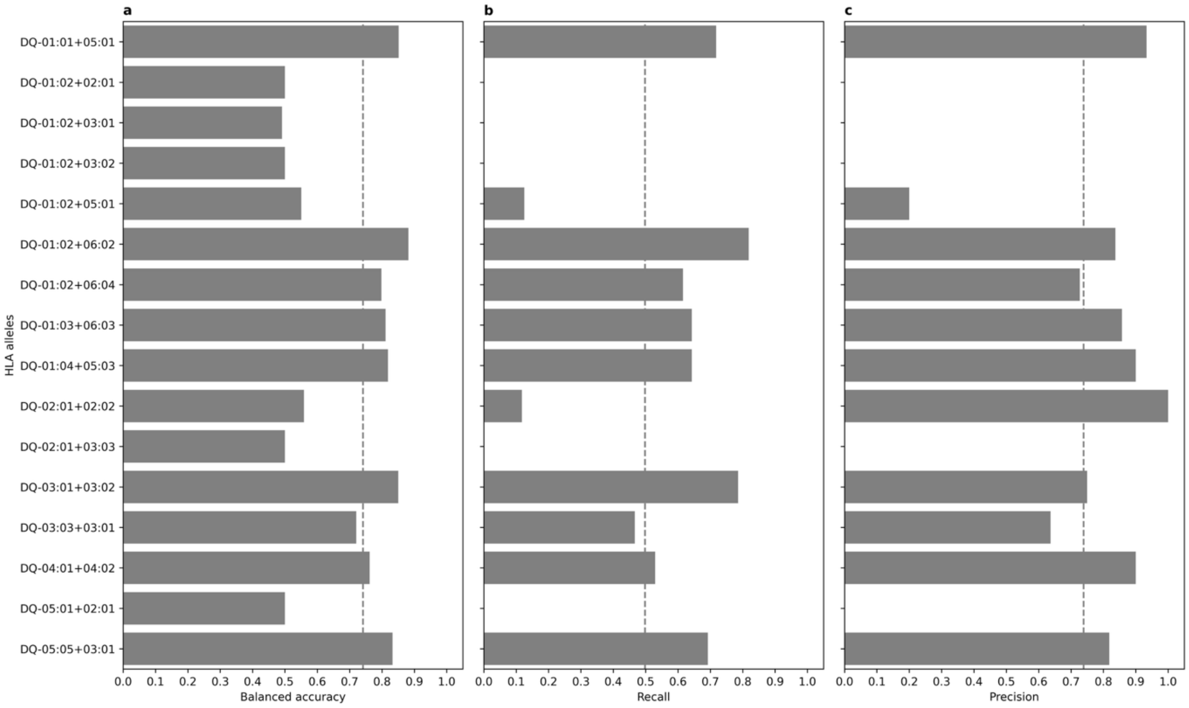

Supplementary Figure 24: *The performance of models to predict the HLA-DQ allele-carriership status from the TRA repertoire evaluated on a test dataset made of 171 repertoires.* These models were trained on a discovery dataset composite of 684 TRA repertoires with matching HLA calls. On the y-axis the models of each *HLA-DQ* allele are shown, for example, DQ-01:01+02:01 represents a model that predicts whether an individual is a carrier for the HLA-DQA1*01:01-DQB1*02:01 allele or not. (**a**), (**b**) and (**c**), depict the balanced accuracy, recall and precision, respectively. Across all panels, grey dashed lines represent the median.

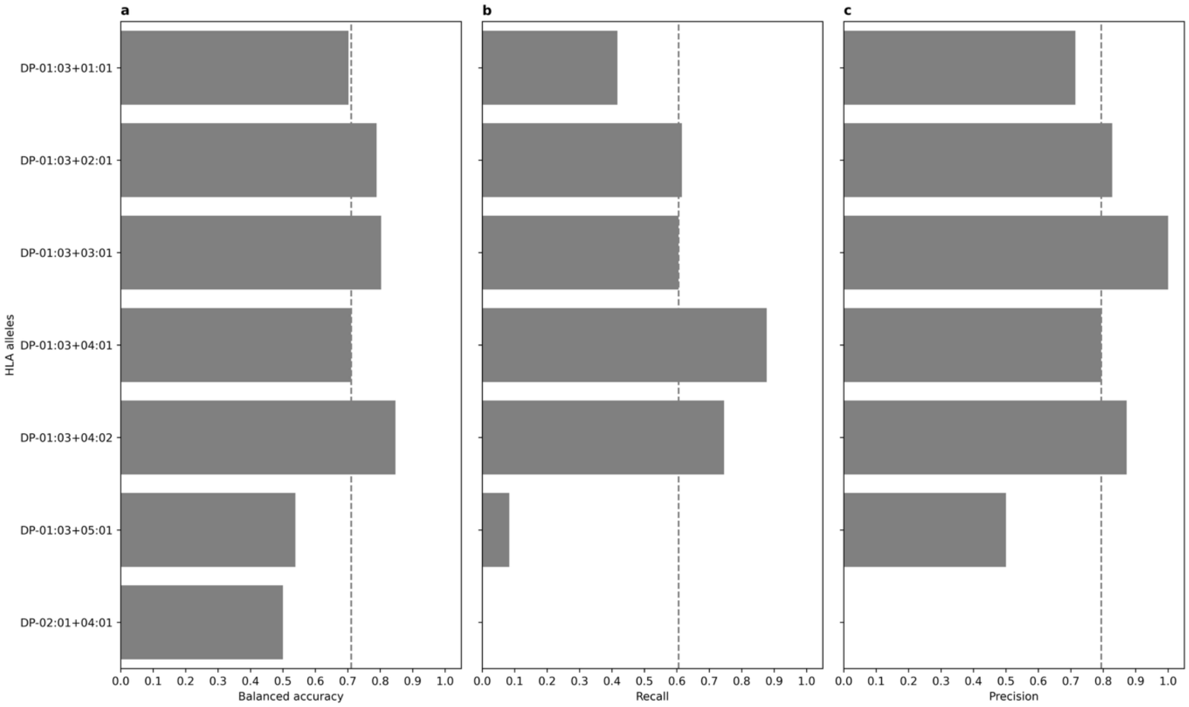

Supplementary Figure 25: *The performance of models to predict the HLA-DP allele-carriership status from the TRA repertoire evaluated on a test dataset made of 171 repertoires.* These models were trained on a discovery dataset composite of 684 TRA repertoires with matching HLA calls. On the y-axis the models of each *HLA-DP* allele are shown, for example, DP-01:03+01:01 represents a model that predicts whether an individual is a carrier for the HLA-DPA1*01:03-DPB1*01:01 allele or not. (**a**), (**b**) and (**c**), depict the balanced accuracy, recall and precision, respectively. Across all panels, grey dashed lines represent the median.

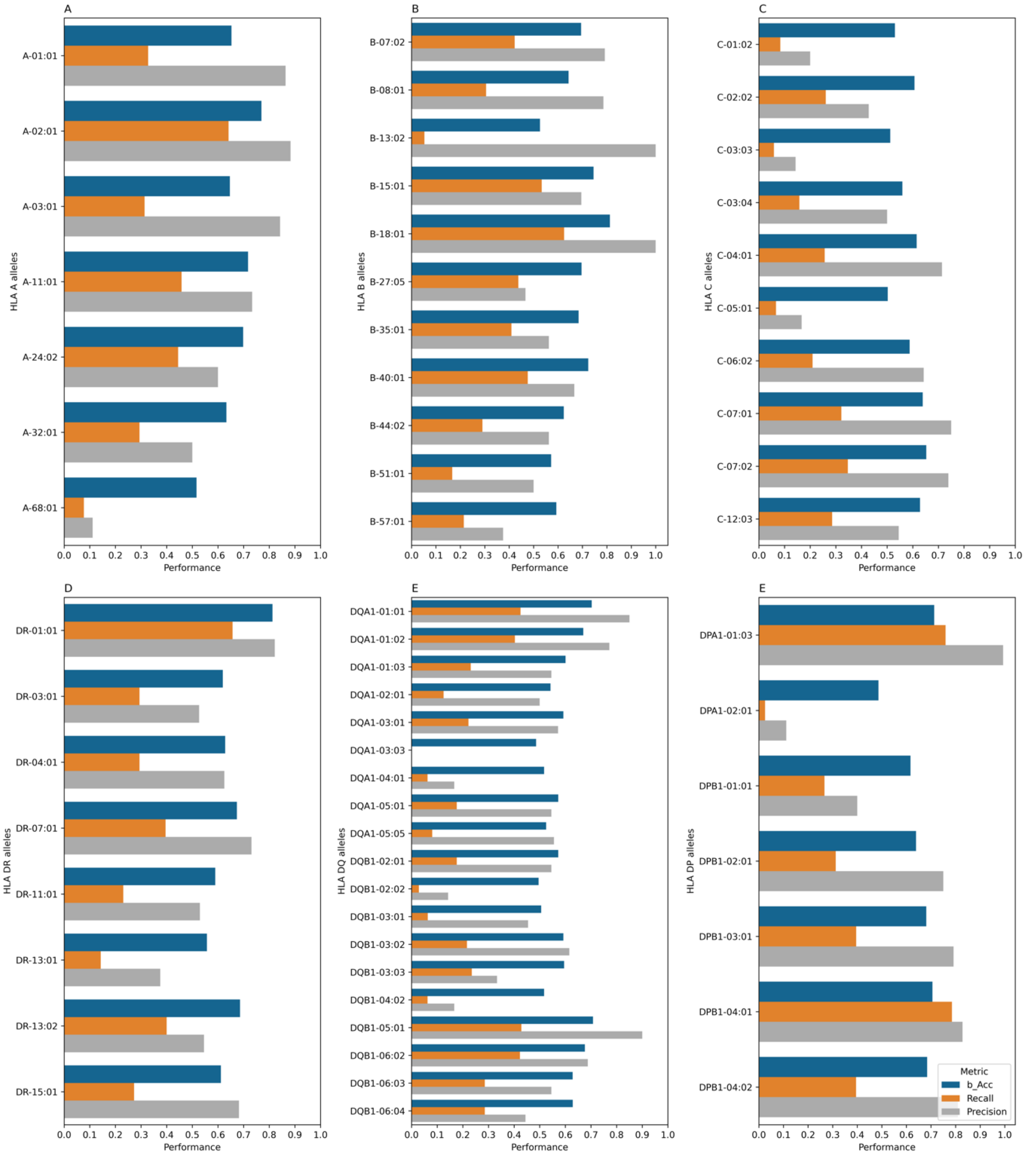

Supplementary Figure 26: T*he performance of the developed TRA-based imputation models on a test dataset of paired TRA repertoire and HLA allotypes generated by Rosati et al.*[1] . (**A-E**) shows the performance on the *HLA-A*, *HLA-B*, *HLA-C*, *HLA-DR*, *HLA-DQA*/*DQB* and *HLA-DPA*/*DPB* alleles, respectively. Across all panels, alleles with carriership frequency <5% (n<12 samples) were excluded from the analysis.

References

1. Rosati, E. *et al.* A novel unconventional T cell population enriched in Crohn's disease. *Gut* 71, 2194 LP – 2204 (2022).
2. Nolan, S. *et al.* A large-scale database of T-cell receptor beta (TCRβ) sequences and binding associations from natural and synthetic exposure to SARS-CoV-2. *Res Sq* (2020).
