# Supplementary material for: T cell receptor clonotypes predict human leukocyte antigen allele carriage and antigen exposure history
Source: Commun Biol. 2026 Jan 13;9:50. doi: 10.1038/s42003-025-09140-2 (PMC12800166; doi:10.1038/s42003-025-09140-2)
Supplement: Supplementary file 3 — description of additional supplementary files [file 42003_2025_9140_MOESM3_ESM.pdf]

Description of Additional Supplementary Files

File name: Supplementary Data 1

Description: The performance of the developed TRB models on the validation dataset.

File name: Supplementary Data 2

Description: The predicted cis and trans HLA-DQ complexes by comparing the expected frequency of these HLA-DQ complexes to their observed frequency using a chi-square test, followed by a Bonferroni correction to label HLA-DQ complexes as potential cis or potential trans HLA-DQ complex.

File name: Supplementary Data 3

Description: The predicted cis and trans HLA-DP complexes by comparing the expected frequency of these HLA-DP complexes to their observed frequency using chi-square test, followed by a Bonferroni correction to label HLA-DP complexes as potential cis or potential trans HLA-DP complex

File name: Supplementary Data 4

Description: The performance of the TRB-based HLA imputation models on an independent test dataset obtained from Rosati *et al.*[24].

File name: Supplementary Data 5

Description: The performance of the TRB-based HLA imputation models on an independent test dataset obtained from the immuneCODE dataset[25].

File name: Supplementary Data 6

Description: The performance of the developed TRA-based imputation models on a test dataset of paired TRA repertoire and HLA alleles that was generated by Rosati et al.[24].

File name: Supplementary Data 7

Description: The predictive performance of HLAGuessr[12] and TCR2HLA on the immuneCODE[25] test dataset**.**
