# Supplementary material for: T cell receptor clonotypes predict human leukocyte antigen allele carriage and antigen exposure history
Source: Commun Biol. 2026 Jan 13;9:50. doi: 10.1038/s42003-025-09140-2 (PMC12800166; doi:10.1038/s42003-025-09140-2)
Supplement: Supplementary file 5 — Reporting summary [file 42003_2025_9140_MOESM5_ESM.pdf]

nature portfolio

Corresponding author(s): Andre Franke; Hesham ElAbd

Last updated by author(s): Jul 23, 2025



# Reporting Summary

Nature Portfolio wishes to improve the reproducibility of the work that we publish. This form provides structure for consistency and transparency in reporting. For further information on Nature Portfolio policies, see our Editorial Policies and the Editorial Policy Checklist.

## Statistics

For all statistical analyses, confirm that the following items are present in the figure legend, table legend, main text, or Methods section.

| n/a | Confirmed | |
|---|---|---|
| ☐ | ☒ | The exact sample size ($n$) for each experimental group/condition, given as a discrete number and unit of measurement |
| ☐ | ☒ | A statement on whether measurements were taken from distinct samples or whether the same sample was measured repeatedly |
| ☐ | ☒ | The statistical test(s) used AND whether they are one- or two-sided<br>*Only common tests should be described solely by name; describe more complex techniques in the Methods section.* |
| ☒ | ☐ | A description of all covariates tested |
| ☒ | ☐ | A description of any assumptions or corrections, such as tests of normality and adjustment for multiple comparisons |
| ☒ | ☐ | A full description of the statistical parameters including central tendency (e.g. means) or other basic estimates (e.g. regression coefficient) AND variation (e.g. standard deviation) or associated estimates of uncertainty (e.g. confidence intervals) |
| ☒ | ☐ | For null hypothesis testing, the test statistic (e.g. $F$, $t$, $r$) with confidence intervals, effect sizes, degrees of freedom and $P$ value noted<br>*Give P values as exact values whenever suitable.* |
| ☒ | ☐ | For Bayesian analysis, information on the choice of priors and Markov chain Monte Carlo settings |
| ☒ | ☐ | For hierarchical and complex designs, identification of the appropriate level for tests and full reporting of outcomes |
| ☒ | ☐ | Estimates of effect sizes (e.g. Cohen's $d$, Pearson's $r$), indicating how they were calculated |

*Our web collection on statistics for biologists contains articles on many of the points above.*

## Software and code

Policy information about availability of computer code

| | |
|---|---|
| Data collection | The paired TCR and HLA genotypes utilized in the current study were collected from multiple previously published studies as depicted in Table 1 in the manuscript. |
| Data analysis | Python 3 were used for implementing and developing the code used for discovering TCR-HLA association and for developing the imputation models. PySpark and Pandas were used for data loading and processing. SciPy were used for performing statistical analyses, scikit-learn and TensorFlow were utilized for developing and implementing the different machine learning models described in the manuscript. Lastly, visualization was conducted using the Matplotlib and seaborn libraries. |

For manuscripts utilizing custom algorithms or software that are central to the research but not yet described in published literature, software must be made available to editors and reviewers. We strongly encourage code deposition in a community repository (e.g. GitHub). See the Nature Portfolio guidelines for submitting code & software for further information.

## Data

Policy information about availability of data

All manuscripts must include a data availability statement. This statement should provide the following information, where applicable:

- Accession codes, unique identifiers, or web links for publicly available datasets
- A description of any restrictions on data availability
- For clinical datasets or third party data, please ensure that the statement adheres to our policy

Due to GDPR and consent restrictions the datasets reported in the current study, namely, the T cell repertoires and paired HLA alleles of German individuals, can be obtained by submitting a project application to the popgen 2.0 Network (https://portal.popgen.de/). Regarding the Norwegian samples, upon contact with Marte Lie Høivik (m.l.hoivik@medisin.uio.no) an institutional data transfer agreement can be established and data shared if the aims of data use are covered by ethical approval and patient consent. The procedure will involve an update to the ethical approval as well as review by legal departments at both institutions, and the process will typically take one to two months from initial contact. Lastly, the US-based datasets are available upon approved application to the Crohn's & Colitis Foundation IBD Plexus Program (https://www.crohnscolitisfoundation.org/ibd-plexus).

## Research involving human participants, their data, or biological material

Policy information about studies with human participants or human data. See also policy information about sex, gender (identity/presentation), and sexual orientation and race, ethnicity and racism.

| | |
|---|---|
| Reporting on sex and gender | Our study investigated the association between the genetically encoded HLA region and the T cell repertoire. Because HLA variation is not influenced by biological sex, and our analyses did not involve sex-dependent variables or stratification, sex was not considered a relevant factor in the study design or analysis. |
| Reporting on race, ethnicity, or other socially relevant groupings | Although most of the cohorts in our study consist of individuals of European ancestry from Norway, Germany, and the United States, we did not perform analyses stratified by self-reported race or genetic ancestry. |
| Population characteristics | As outlined in Table 1, our study includes multiple cohorts composed of individuals with inflammatory bowel disease (IBD), primary sclerosing cholangitis (PSC), or healthy controls. The phenotypic characteristics of these cohorts are detailed in their respective source studies, as cited in Table 1. |
| Recruitment | We did not conduct active participant recruitment; instead, we used previously published datasets as the starting material for our analysis of TCR–HLA associations. |
| Ethics oversight | The study has been approved by the ethical committee at the University of Kiel under the following ethical votes: D441/16, D474/12, A161/08, A103/14, and A148/14. For the Norwegian cohort, namely, H3, CD4, and UC4 datasets were derived from the IBSEN III study which was approved by the South-Eastern Regional Committee for Medical and Health Research Ethics (Ref 2015/946-3) and performed in accordance with the Declaration of Helsinki. The USA-based samples, namely, UC2 and CD2 are derived from the SPARC IBD cohort from the IBD Plexus research program maintained by the Crohn's & Colitis Foundation and described by Raffals et al.50 A written informed consent was collected from all participants prior to the beginning of the study. |

Note that full information on the approval of the study protocol must also be provided in the manuscript.

# Field-specific reporting

Please select the one below that is the best fit for your research. If you are not sure, read the appropriate sections before making your selection.

☒ Life sciences ☐ Behavioural & social sciences ☐ Ecological, evolutionary & environmental sciences

For a reference copy of the document with all sections, see nature.com/documents/nr-reporting-summary-flat.pdf

# Life sciences study design

All studies must disclose on these points even when the disclosure is negative.

| | |
|---|---|
| Sample size | We assembled a large dataset comprising 6,794 paired HLA allotypes and TCR repertoires. |
| Data exclusions | We did not exclude any data points initially; however, in downstream analyses, we focused on HLA alleles with a carriership frequency of at least 1% or 5%, depending on the specific analysis and dataset. |
| Replication | We replicated our findings using two previously published datasets: the immuneCODE dataset described by Nolan et al. (2020) and the dataset reported by Rosati et al. (2022). |
| Randomization | Randomization was not applicable to this study, as we performed retrospective TCR–HLA association analyses using previously published datasets without assigning participants to experimental groups. |

| Blinding | Blinding was not applicable, as our study involved computational analysis of previously published datasets without experimental group assignment or subjective outcome assessment. |
|---|---|

# Reporting for specific materials, systems and methods

We require information from authors about some types of materials, experimental systems and methods used in many studies. Here, indicate whether each material, system or method listed is relevant to your study. If you are not sure if a list item applies to your research, read the appropriate section before selecting a response.

## Materials & experimental systems

| n/a | Involved in the study |
|---|---|
| ☒ | ☐ Antibodies |
| ☒ | ☐ Eukaryotic cell lines |
| ☒ | ☐ Palaeontology and archaeology |
| ☒ | ☐ Animals and other organisms |
| ☒ | ☐ Clinical data |
| ☒ | ☐ Dual use research of concern |
| ☒ | ☐ Plants |

## Methods

| n/a | Involved in the study |
|---|---|
| ☒ | ☐ ChIP-seq |
| ☒ | ☐ Flow cytometry |
| ☒ | ☐ MRI-based neuroimaging |

## Plants

| | |
|---|---|
| Seed stocks | *Report on the source of all seed stocks or other plant material used. If applicable, state the seed stock centre and catalogue number. If plant specimens were collected from the field, describe the collection location, date and sampling procedures.* |
| Novel plant genotypes | *Describe the methods by which all novel plant genotypes were produced. This includes those generated by transgenic approaches, gene editing, chemical/radiation-based mutagenesis and hybridization. For transgenic lines, describe the transformation method, the number of independent lines analyzed and the generation upon which experiments were performed. For gene-edited lines, describe the editor used, the endogenous sequence targeted for editing, the targeting guide RNA sequence (if applicable) and how the editor was applied.* |
| Authentication | *Describe any authentication procedures for each seed stock used or novel genotype generated. Describe any experiments used to assess the effect of a mutation and, where applicable, how potential secondary effects (e.g. second site T-DNA insertions, mosiacism, off-target gene editing) were examined.* |
